# Calcium in the Pathophysiology of Atrial Fibrillation and Heart Failure

**DOI:** 10.3389/fphys.2018.01380

**Published:** 2018-10-04

**Authors:** Nathan C. Denham, Charles M. Pearman, Jessica L. Caldwell, George W. P. Madders, David A. Eisner, Andrew W. Trafford, Katharine M. Dibb

**Affiliations:** Unit of Cardiac Physiology, Division of Cardiovascular Sciences, Manchester Academic Health Science Centre, University of Manchester, Manchester, United Kingdom

**Keywords:** atrial fibrillation, pathophysiology, calcium, heart failure, t-tubules

## Abstract

Atrial fibrillation (AF) is commonly associated with heart failure. A bidirectional relationship exists between the two—AF exacerbates heart failure causing a significant increase in heart failure symptoms, admissions to hospital and cardiovascular death, while pathological remodeling of the atria as a result of heart failure increases the risk of AF. A comprehensive understanding of the pathophysiology of AF is essential if we are to break this vicious circle. In this review, the latest evidence will be presented showing a fundamental role for calcium in both the induction and maintenance of AF. After outlining atrial electrophysiology and calcium handling, the role of calcium-dependent afterdepolarizations and atrial repolarization alternans in triggering AF will be considered. The atrial response to rapid stimulation will be discussed, including the short-term protection from calcium overload in the form of calcium signaling silencing and the eventual progression to diastolic calcium leak causing afterdepolarizations and the development of an electrical substrate that perpetuates AF. The role of calcium in the bidirectional relationship between heart failure and AF will then be covered. The effects of heart failure on atrial calcium handling that promote AF will be reviewed, including effects on both atrial myocytes and the pulmonary veins, before the aspects of AF which exacerbate heart failure are discussed. Finally, the limitations of human and animal studies will be explored allowing contextualization of what are sometimes discordant results.

## Introduction

Two highly prevalent forms of cardiovascular disease are atrial fibrillation (AF) and heart failure, and in spite of recent advances in treatment these conditions remain important causes of morbidity and mortality. AF is an abnormal heart rhythm affecting more than 30 million patients worldwide (Chugh et al., 2014), and is characterized by rapid and disorganized electrical activity within the cardiac atria (Kirchhof et al., 2016). This results in the loss of atrial contraction, irregular ventricular contractions, and has a detrimental effect on the lives of those who suffer from it ranging from a reduction in day-to-day quality of life secondary to symptoms such as palpitations and exercise intolerance (Thrall et al., 2006), and an increased risk of heart failure (Stewart et al., 2002), stroke (Wolf et al., 1991), and premature death (Benjamin et al., 1998). Heart failure is defined as the presence of symptoms such as breathlessness resulting from cardiac structural or functional abnormalities that in general cause impaired contraction and/or relaxation of the myocardium (Ponikowski et al., 2016). This life-threatening condition affects 1–2% of the general population in the developed world (Mosterd and Hoes, 2007), and carries higher mortality rates than many cancers (Mamas et al., 2017). Of increasing importance is the bidirectional relationship between AF and heart failure. To put it simply, those with AF are more likely to develop heart failure and vice versa.

Heart failure occurs in up to one third of patients with AF (Santhanakrishnan et al., 2016), which may be as a direct result of rapid ventricular rates in AF [known as a tachycardia-induced cardiomyopathy (Fujino et al., 2007)] or the association of risk factors common to both conditions such as hypertension (Benjamin et al., 1994; Levy et al., 1996). If heart failure occurs in those with AF, the patient is likely to face an increased burden of symptoms, more admissions to hospital and a lower chance of restoring sinus rhythm (Silva-Cardoso et al., 2013; Odutayo et al., 2017). On the other hand, in those who initially have a normal cardiac rhythm, heart failure is associated with a 5-fold increase in the risk of developing new AF (Benjamin et al., 1994) due to electrical and structural remodeling in the atria (Nattel et al., 2007; Nattel and Harada, 2014), which can also increase rates of hospitalization, stroke, and death (Dries et al., 1998; McManus et al., 2013; Odutayo et al., 2017).

Disordered calcium handling is a key link in the bidirectional relationship between AF and heart failure and it is the aim of this review to provide the reader with an up-to-date overview of this important topic. We will start by exploring the role of calcium in excitation-contraction coupling in healthy atria followed by an overview of the general mechanisms of arrhythmias. While the effects of AF on calcium handling have been reviewed recently e.g., Dobrev and Wehrens (2017) and Landstrom et al. (2017), here we focus firstly on the pathological remodeling of calcium handling which determines the progression of AF from short-lived paroxysms to longer durations of persistent AF. To achieve this, we have attempted to stratify studies in terms of the stage of AF (from early remodeling to persistent AF). Secondly, we examine how heart failure promotes AF with an emphasis on calcium cycling in the atria, providing an insight into the fundamental changes which drive both conditions, including the role played by t-tubules. In addition, we describe the mechanisms by which AF exacerbates heart failure, and how a vicious circle can be generated. Finally, this review provides a critical appraisal of both human and animal studies in this field, to highlight limitations that should influence future research.

## Calcium, excitation-contraction coupling, and arrhythmias

To appreciate the association between AF, heart failure, and disturbances in calcium handling, we must first understand the role of calcium in excitation contraction coupling in healthy atrial tissue. The action potential is initiated by the rapid influx of sodium (*I*_Na_) resulting in membrane depolarization. The shape of the atrial action potential differs from that of the ventricle, at least in larger species, where generally the atrial action potential is shorter and more triangulated (Hume and Uehara, 1985; Giles and Imaizumi, 1988; Amos et al., 1996; Xu et al., 1999) as reviewed in Nerbonne and Kass (2005). Depolarization activates L-type calcium channels (LTCCs) allowing a small amount of calcium to enter the cell. (*I*_Ca(L)_) (Bers, 2002; Eisner et al., 2017).

**Figure 1:**
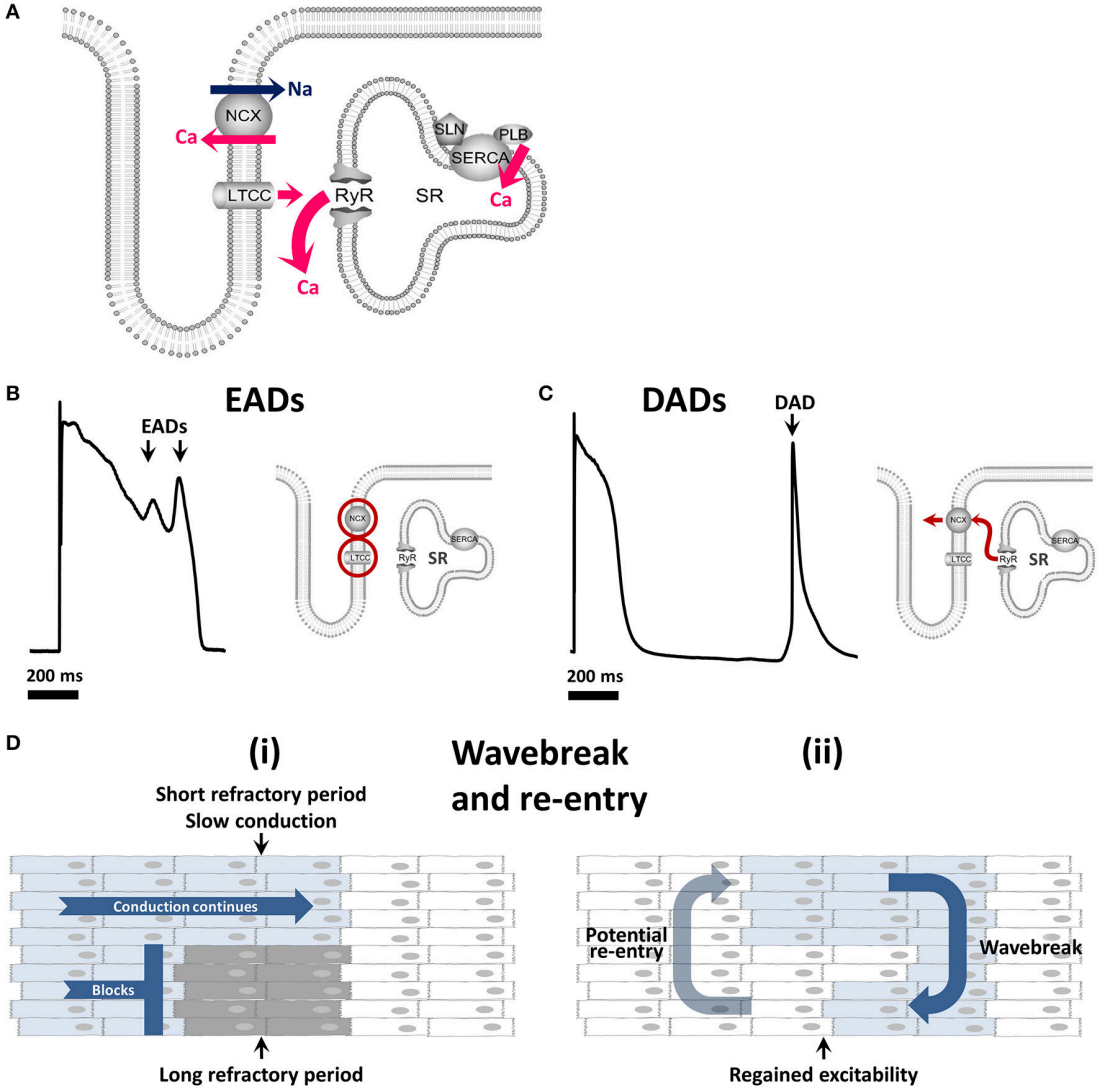
Calcium cycling and mechanisms of arrhythmia. **(A)** The role of the calcium cycling mechanism in calcium induced calcium release **(B)** Early afterdepolarizations arise through reactivation of *I*_Ca(L)_ and / or *I*_Na_ and are facilitated by *I*_NCX_. **(C)** Delayed afterdepolarizations arise through spontaneous calcium release from the sarcoplasmic reticulum. **(D)** (i) Wavebreak occurs when a smooth depolarizing wave front encounters an area of inexcitable tissue (ii) which may progress to re-entry whereby the depolarizing wave continues to rotate around an inexcitable core. EAD, early afterdepolarization; DAD, delayed afterdepolarization; LTCC, L-type calcium channel; RyR, ryanodine receptor; SR, sarcoplasmic reticulum; NCX, sodium/calcium exchanger; SERCA, sarco-endoplasmic reticulum calcium ATPase.

As in ventricular myocytes, a small influx of calcium via *I*_Ca(L)_ triggers the release of a larger quantity of calcium from the sarcoplasmic reticulum (SR) into the cytoplasm via ryanodine receptors (RyRs) in a process known as calcium-induced calcium release (CICR; Figure 1A) (Bers, 2002; Eisner et al., 2017). It is important to mention that although this process is mechanistically similar, the relative contributions of the individual components differ (Walden et al., 2009).

CICR is rapid and in the healthy ventricle occurs near-simultaneously throughout the cell due to two main factors. Firstly, LTCCs on the cell membrane and RyRs on the SR membrane associate closely to form dyads allowing efficient triggering of calcium release. Secondly, deep invaginations of the surface membrane known as transverse (t)-tubules allow dyads to occur not only at the cell periphery but throughout the cell interior, such that calcium rises in a more synchronous manner in ventricular myocytes (Figure 1A). Calcium release is not completely synchronous in atrial cells as there are fewer t-tubules than in ventricular myocytes (Dibb et al., 2009; Caldwell et al., 2014), and their abundance changes with cell width, species, and location in the atria (Richards et al., 2011; Frisk et al., 2014; Glukhov et al., 2015; Gadeberg et al., 2016). These factors likely contribute to the diversity of atrial t-tubule density reported, particularly in small mammalian species, where t-tubules are either lacking (Huser et al., 1996; Brette et al., 2002), sparse, and often longitudinally orientated (Kirk et al., 2003; Woo et al., 2005) or can be more abundant (Frisk et al., 2014; Glukhov et al., 2015). It is generally well established that atrial t-tubules are common in large mammalian species e.g., sheep, cow, horse, dog, and pig although t-tubule levels are heterogeneous between cells and species (Wakili et al., 2010; Richards et al., 2011; Frisk et al., 2014; Gadeberg et al., 2016). Since many of these species are used as models of AF it is important that atrial t-tubule levels mimic those found in the human. T-tubules have been shown to be present to some extent in almost 70% of human atrial cells (Richards et al., 2011) although only sparse t-tubules have been reported in isolated human atrial cells (Greiser et al., 2014). Differences between studies may arise due to t-tubule damage, suggested to occur as a result of enzymatic digestion (Chen et al., 2015), or to differences in the underlying pathophysiology of patient samples and therefore the reliance of systolic calcium on human atrial t-tubules is not well understood.

Following the release of calcium from the SR, free intracellular calcium is then available to bind to troponin C, causing myocyte contraction. Relaxation is brought about by the removal of calcium from the cytosol which occurs via two main mechanisms: reuptake into the SR by the sarcoplasmic reticulum calcium ATPase pump (SERCA) regulated by the inhibitory proteins phospholamban and sarcolipin, and removal from the cell via the sodium-calcium exchanger (NCX) on the plasma membrane (Figure 1A). Minor contributions to calcium removal come from the plasma membrane calcium ATPase pump and the mitochondrial calcium uniporter. When free cytoplasmic calcium falls, it dissociates from troponin C, bringing about relaxation during the diastolic phase of the action potential (phase 4) (Bers, 2001).

In the steady state, calcium influx and efflux are maintained in balance. Any increase in calcium influx e.g., during beta adrenergic stimulation, is met by a corresponding increase in calcium efflux (Trafford et al., 1997; Eisner et al., 2013). Under normal conditions, this ensures cells do not become overloaded with calcium. SR calcium content can be increased but only if influx exceeds efflux until a new steady state of influx and efflux is achieved (Trafford et al., 1997). In conditions such as AF and heart failure, pathological remodeling of calcium handling can promote arrhythmias.

### The basis of arrhythmias

Like all tachyarrhythmias, the onset and maintenance of AF is dependent on three key mechanisms: automaticity, triggered activity, and re-entry (Antzelevitch and Burashnikov, 2011). Automaticity, whereby an excitable tissue spontaneously depolarizes, is an inherent property of the specialized tissue within the cardiac conduction system. Abnormal automaticity occurs when an area outside the conduction system becomes capable of automatic activity faster than the leading pacemaker site, or less commonly when disease accelerates the firing rate of nodal tissue.

Triggered activity refers to additional impulses triggered by an initial action potential, known as afterdepolarizations. Although several mechanisms can lead to afterdepolarizations, many of these relate to abnormalities in calcium handling. There are two types of afterdepolarization termed “early” or “delayed” afterdepolarizations. Early afterdepolarizations (EADs; Figure 1B), occur within the action potential (phases 2–3) and may arise from reactivation of *I*_Ca(L)_ or spontaneous calcium release (Qi et al., 2009) driving reverse mode *I*_NCX_ as reviewed by Weiss et al. (2010). Delayed afterdepolarizations (DADs; Figure 1C), occurring after the action potential (within phase 4) arise from spontaneous calcium release from the SR in the form of a calcium wave (Venetucci et al., 2008). This spontaneous calcium transient leaves the cell via NCX in exchange for sodium, creating a transient inward current (*I*_ti_). If the depolarizations induced by EADs or DADs are large enough they may lead to one or more additional premature action potentials.

Both normal and arrhythmogenic calcium release from the SR occurs via RyRs. Calcium sparks are the elementary calcium release events of the SR which occur when RyRs within a cluster open (Cheng et al., 1993; Rajagopal et al., 2015; Walker et al., 2015). Under conditions of increased calcium loading, spark frequency increases and calcium sparks are able to trigger propagating calcium waves (Cheng et al., 1993). Therefore calcium waves occur when the SR reaches a threshold calcium content (Diaz et al., 1997; Venetucci et al., 2007). The calcium release kinetics of the RyR can be modified by phosphorylation at the protein kinase A site or at the CaMKII site resulting in increased RyR calcium sensitivity and open probability (Wehrens et al., 2004; Vest et al., 2005; Huke and Bers, 2008). In addition, accessory proteins such as junctophilin (Beavers et al., 2013), FKBP-12.6 (Marx et al., 2000), calsequestrin, triadin, and junctin (Gyorke et al., 2004) can all modulate RyR open probability.

The third main arrhythmic mechanism is re-entry whereby waves of depolarization circle around an obstacle, continuously reinitiating in the manner of a dog chasing its tail (Figure 1D). This obstacle may be anatomical, such as scar tissue, but functional obstacles such as a core of tissue rendered inexcitable by continuous depolarization can also permit re-entry. This functional re-entry is of particular importance in AF (Allessie et al., 1977; Comtois et al., 2005).

These fundamental mechanisms feed into several models of AF including the classical model of multiple re-entrant circuits continually forming and extinguishing (Allessie et al., 1996) and the more recent view of one or more primary re-entrant drivers or rotors which break down to fibrillatory conduction at the peripheries (Berenfeld and Jalife, 2014). A discussion on the merits of these models can be found elsewhere (Allessie and de Groot, 2014; Narayan and Jalife, 2014).

## The pathophysiology of atrial fibrillation

### The natural history of atrial fibrillation

In many patients who are otherwise free from cardiovascular disease, AF is first seen as short bursts of fibrillation that last from minutes to hours before reverting spontaneously to a normal sinus rhythm in a pattern known as paroxysmal AF (Kirchhof et al., 2016). These bursts are initiated by electrical impulses known as triggers, most commonly arising from the pulmonary veins (Haissaguerre et al., 1998). Over time, the frequency and duration of AF tends to increase until eventually the arrhythmia becomes both continuous and self-sustaining (Gillis and Rose, 2000), and is known as persistent AF. The electrical properties and structure of the atria that determine how well the atria support AF are together known as the atrial substrate. The increasing tendency to AF occurs due to changes in the atrial substrate brought about by the rapid stimulation of the atria during bouts of AF, and are summed up by the phrase “AF begets AF” (Wijffels et al., 1995). However, the stepwise progression from paroxysmal to persistent AF is not universal, as those who have structural heart disease such as heart failure may have developed a pro-arrhythmic substrate before AF arises, and may therefore immediately present with persistent AF (Leclercq et al., 2014). Dysregulation of calcium is involved both in the triggering of AF, and the progression of the atrial substrate that facilitates AF. In this section, we aim to describe the time course of key changes in calcium cycling with regard to the stage of AF, highlighting not only how “calcium cycling remodeling begets further remodeling” but what may drive the atria down this path rather than be bystander effects.

### What triggers atrial fibrillation?

In patients with “lone AF,” the early stages of the disease are predominantly due to abnormally frequent triggering impulses. In humans, the commonest sites for triggers are the pulmonary veins from which rapid electrical activity can both initiate and sustain AF (Oral et al., 2002). The pulmonary veins are estimated to be the source of triggers in ~ 90% of patients with paroxysmal AF. This has led to the development of ablation therapy which aims to electrically isolate the pulmonary veins from the left atrium and thereby prevent triggers from reaching the myocardium (Haissaguerre et al., 1998). Although triggers can arise from other areas of the atria such as the left atrial appendage (Santangeli et al., 2016), the key role of the pulmonary vein sleeves is supported by mechanistic studies demonstrating features that predispose these tissues to abnormal firing (Arora et al., 2003).

The myocytes which extend up the pulmonary vein sleeves can fire independently (Perez-Lugones et al., 2003), potentially due to automaticity, triggered activity, and micro re-entry [as reviewed in Takahara et al. (2014)]. These myocytes are electrophysiologically distinct from those in the atria. For example canine pulmonary vein sleeves display both decreased *I*_K1_ and *I*_Ca(L)_ but increased delayed rectifier currents compared to atrial myocardium (Ehrlich et al., 2003). The net effect of these differences is a shorter action potential duration and less negative resting membrane potential which together facilitate calcium-dependent afterdepolarizations and triggered activity (Patterson et al., 2006).

The automaticity and triggered activity seen in pulmonary vein myocytes may be calcium dependent, as some studies report asynchronous spontaneous calcium transients in myocytes in intact pulmonary veins (Chou et al., 2005; Logantha et al., 2010; Rietdorf et al., 2014). Some have found that these spontaneously active cells have higher SR calcium content and a corresponding increase in the frequency of calcium sparks compared to atrial myocytes which could contribute to these spontaneous events (Chang et al., 2008). However, spontaneous pulmonary vein activity has not been found in all studies, and the calcium handling properties of canine pulmonary vein myocytes are generally unaltered compared to those from the atria (Hocini et al., 2002; Perez-Lugones et al., 2003; Coutu et al., 2006). Increased firing from the veins has been reported during periods of tachycardia (Chen et al., 2000; Honjo et al., 2003) and during rapid changes in autonomic tone (Zimmermann and Kalusche, 2001; Tomita et al., 2003; Patterson et al., 2005) due to an increase in the predisposition to calcium-dependent afterdepolarizations at these times.

In addition to the electrophysiological and calcium handling differences, the anatomy of the veins also promotes re-entry due to the combination of abrupt changes in fiber orientation and reduced electrical connectivity between muscle bundles creating areas of heterogeneous conduction velocity and localized block (Hocini et al., 2002; Arora et al., 2003). Calcium may also play a role in the propagation of triggered impulses from the pulmonary veins to the atria via small conductance calcium-activated potassium channels. Rapid stimulation increases the expression of these channels and shortens the action potential in the pulmonary veins. However, the effect of blocking these channels is unclear as both pro-arrhythmic (Hsueh et al., 2013) and antiarrhythmic effects (Ozgen et al., 2007; Qi et al., 2014) have been described.

Taken together, the available evidence supports a greater propensity for the pulmonary veins to generate spontaneous activity although the precise mechanism is controversial.

### What makes an atrial substrate vulnerable to atrial fibrillation?

The atrial substrate refers to the sum of all atrial characteristics that influence how readily the atria support fibrillation. In response to triggering impulses, a healthy atrial substrate may generate fibrillation that extinguishes after a very short period, or may not support fibrillation at all. On the other hand, a diseased, remodeled and thence vulnerable atrial substrate will often manifest prolonged fibrillation in response to the same triggering impulses (Sanders et al., 2003; Cha et al., 2004a; Todd et al., 2004). The atrial substrate changes over time and in response to high atrial rates as seen during fibrillation, in part mediated by intracellular calcium.

Many of the characteristics that contribute to the vulnerability of the atrial substrate can be understood using the classical multiple-wavelet paradigm of AF in which AF results from many wavelets following re-entrant circuits that continuously form and extinguish in a chaotic manner (Moe et al., 1964). The size of these wavelets is a product of the conduction velocity of the tissue and the effective refractory period (ERP), referred to as the wavelength. The atrial ERP is primarily determined by the action potential duration (Bode et al., 2001). Atrial tissue with properties that support a shorter wavelength exhibit wavelets that rotate around a smaller volume of tissue (Zou et al., 2005). In this model, AF terminates when all wavelets have extinguished at a single time-point, which becomes increasingly unlikely the more wavelets the atria can sustain.

Structural aspects of the atria therefore influence atrial vulnerability. Larger atria as found in heart failure can sustain more wavelets simultaneously, and are therefore more vulnerable to AF (Sridhar et al., 2009; Melenovsky et al., 2015). Fibrosed atria, arising as a consequence of heart failure (Li et al., 1999) or obesity (Fukui et al., 2013), conduct electrical impulses more slowly, leading to a shorter wavelength and allowing more re-entrant circuits to co-exist in a given mass of tissue (Van Wagoner and Nerbonne, 2000). Electrophysiological properties such as the atrial refractory period also feed into to this model as a shorter action potential (and thus shorter refractory period) leads to a shorter wavelength permitting a greater number of re-entrant wavelets and facilitating AF. Mechanisms to shorten action potential duration include a decrease in *I*_Ca(L)_, decreased forward mode *I*_NCX_ or an increase in potassium currents (Johannsson and Wohlfart, 1980; Fermini and Schanne, 1991; Viswanathan et al., 1999; Armoundas et al., 2003). To generate these wavelets, the normally smooth and regular waves of depolarization traversing the atria must be disrupted (Zhao et al., 2013). Wavebreak occurs when a wavefront can pass in one region of tissue but is blocked in an adjacent region, causing the wavefront to begin to rotate. This wavebreak is more likely to occur if the refractory periods of the atrial tissue differ markedly from region to region, referred to as a dispersion of repolarization (Allessie et al., 1977). While this dispersion can be fixed, an important dynamic determinant of the dispersion of repolarization is action potential alternans, often driven by calcium dynamics.

Action potential alternans refers to a phenomenon whereby a single cell or region of tissue generates action potentials in a repeated long-short-long-short pattern when stimulated, as reviewed by (Weiss et al., 2011). As this alternating sequence passes through the myocardial tissue, it creates a discordant pattern whereby at a single point in time the action potentials in some regions will be short but in neighboring regions will be long. If a premature triggering impulse occurs, it may potentially block in regions with long action potentials but continue to conduct where action potentials are short, creating wavebreak and re-entry. In this context therefore atrial alternans acts as a substrate for AF. Although action potential alternans can arise from properties of the sarcolemmal ion channels, it can also be driven by instabilities in calcium handling (Eisner et al., 2005). Alternating calcium transient amplitudes can occur due to high stimulation rates, elevated SR calcium content, and alterations to RyR refractoriness, as reviewed by Qu et al. (2013). These then lead to alternation of the action potential: large transients increase calcium-dependent inactivation of *I*_Ca(L)_ and enhance calcium efflux through NCX, simultaneously shortening the plateau of the action potential and extending terminal repolarisation, while small calcium transients produce a longer plateau but shorter terminal repolarization. Atrial alternans can be measured in patients using standard electrophysiological catheters and correlates with vulnerability to AF (Verrier et al., 2016). It has been shown to track the progression of AF in a sheep tachypacing model, to precede the transition from atrial flutter to fibrillation in man, and to occur more frequently in the atria of patients with AF than those without (Narayan et al., 2002, 2008, 2011; Monigatti-Tenkorang et al., 2014).

Another calcium-dependent aspect of the vulnerability of the atrial substrate to AF is calcium loading of the cell during brief periods of rapid atrial stimulation. When the atria are exposed to rapid rates such as triggers from the pulmonary veins, or artificially from rapid pacing, there is a net influx of calcium into the cell via the more frequently activated *I*_Ca(L)_ (Sun et al., 2001). This is presumably primarily driven by the increased stimulation rate increasing influx per unit time because with increasing rates, calcium entry via *I*_Ca(L)_ decreases per beat (Dibb et al., 2007). This increased influx of calcium leads to increased reuptake via SERCA, loading the SR. A new steady state of calcium flux is created at a higher SR calcium content when an increase in the fractional release of calcium during the transient enables sufficient efflux to balance the increased influx. The higher SR calcium content also increases the frequency of RyR opening during diastole promoting a greater *I*_ti_ and increasing the probability of inducing an afterdepolarization (Cheng, H. et al., 1996). Triggers therefore produce immediate pro-arrhythmogenic changes in the atrium encouraging calcium-dependent afterdepolarizations (Ferrier et al., 1973; Terracciano et al., 1995; Santiago et al., 2013) leading to additional action potentials (Burashnikov and Antzelevitch, 2003), further increasing the atrial rate and calcium influx into the cell.

**Figure 2:**
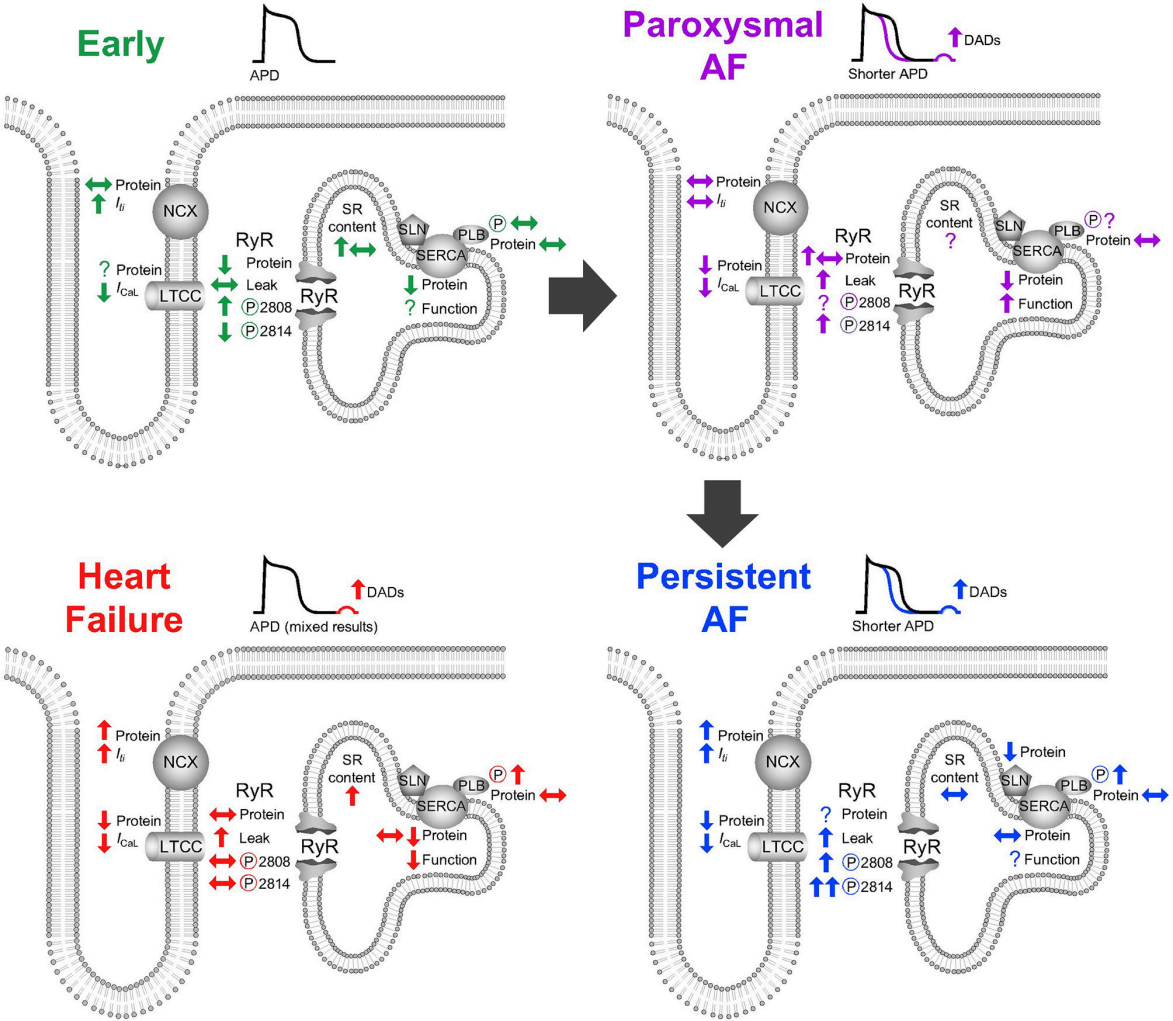
Alterations to atrial calcium handling (top left) in response to short-term rapid atrial stimulation, (top right) in paroxysmal atrial fibrillation, (bottom right) in persistent atrial fibrillation, (bottom left) and in heart failure. APD, action potential duration; DAD, delayed afterdepolarization; LTCC, L-type calcium channel; RyR, ryanodine receptor; SR, sarcoplasmic reticulum; NCX, sodium/calcium exchanger; SERCA, sarco-endoplasmic reticulum calcium ATPase; SLN, sarcolipin; PLB, phospholamban.

Calcium handling is additionally remodeled as a consequence of t-tubule loss which is evident following 7 days of atrial pacing (Wakili et al., 2010) (classified in Table 1 as paroxysmal AF), during persistent AF (Lenaerts et al., 2009) and during heart failure induced by both rapid ventricular pacing (Dibb et al., 2009) and myocardial infarction (Kettlewell et al., 2013). An overview of all changes can be observed in Figure 2. While t-tubule loss occurs as a consequence of AF, it may also either facilitate AF occurrence in heart failure or arrhythmia progression (in the absence of heart failure) by a number of the mechanisms discussed above. Electrically, t-tubule loss, at least in the ventricle, decreases the duration of the action potential (Brette et al., 2006) and if a similar phenomenon occurs in the atria then t-tubule loss may exacerbate action potential shortening in AF and shorten the atrial refractory period. Loss of t-tubular *I*_Ca(L)_ likely contributes to action potential shortening (Brette et al., 2006) and also promotes the decrease in *I*_Ca(L)_ (Lenaerts et al., 2009) which is a hallmark feature of AF.

**Table 1:** A table showing all studies which have investigated changes in calcium cycling in the atrium in either the various stages of atrial fibrillation (**AF**) or in heart failure with a reduced ejection fraction.

		**Early**	**pAF**	**PerAF**	**Heart failure**
Sarcoplasmic Reticulum Calcium Content		↑ Sun et al., 2001 ↔ Greiser et al., 2014	↑ Voigt et al., 2014 ↔ Hove-Madsen et al., 2004 ↓ Greiser et al., 2009; Wakili et al., 2010	↔ Kneller et al., 2002; Lenaerts et al., 2009; Neef et al., 2010; Voigt et al., 2012; Macquaide et al., 2015	↑ Yeh et al., 2008; Clarke et al., 2015; Aistrup et al., 2017 ↓ Saba et al., 2005; Johnsen et al., 2013
LTCC and *I*_Ca(L)_	*α1 subunit mRNA*	↓ Bosch et al., 2003	↓ Brundel et al., 2001	↓ Brundel et al., 1999; Lai et al., 1999; Van Gelder et al., 1999; Yue et al., 1999; van der Velden et al., 2000b; Gaborit et al., 2005	–
			↔ Brundel et al., 1999		
	*α1 subunit protein expression*	–	↓ Brundel et al., 2001; Lugenbiel et al., 2015	↓ Brundel et al., 1999; Klein et al., 2003; Lenaerts et al., 2009	↔ Ouadid et al., 1995; Boixel et al., 2001
			↔ Brundel et al., 1999	↔ Schotten et al., 2003b; Christ et al., 2004	
				↑ Dai et al., 2016	
	*I_*Ca*(*L*)_ density*	↓ Bosch et al., 2003; Qi et al., 2008; Greiser et al., 2014	↓ Yagi et al., 2002; Cha et al., 2004a; Wakili et al., 2010	↓ Yue et al., 1997; Van Wagoner et al., 1999; Workman et al., 2001; Yagi et al., 2002; Christ et al., 2004; Lenaerts et al., 2009; Voigt et al., 2012	↓ Ouadid et al., 1995; Li et al., 2000; Boixel et al., 2001; Cha et al., 2004a,b; Dinanian et al., 2008; Sridhar et al., 2009; Clarke et al., 2015
			↔ Voigt et al., 2014	↑ (single channel only) Klein et al., 2003	↔ Cheng T. H. et al., 1996; Workman et al., 2009
SERCA and accessory proteins	*SERCA2A mRNA*	–	↔ Brundel et al., 1999	↓ Brundel et al., 1999; Lai et al., 1999; Ohkusa et al., 1999; Cao et al., 2002	–
				↔ Van Gelder et al., 1999	
	*SERCA protein expression*	↓ Greiser et al., 2014	↓ Voigt et al., 2014; Lugenbiel et al., 2015; Wang et al., 2017	↔ Hoit et al., 2002; Schotten et al., 2002; El-Armouche et al., 2006; Lenaerts et al., 2009; Neef et al., 2010; Dai et al., 2016	↔ Shanmugam et al., 2011; Clarke et al., 2015
			↔ Brundel et al., 1999; Wakili et al., 2010	↓ Brundel et al., 1999	↓ Yeh et al., 2008
	*SERCA function*	–	↑ Xie et al., 2012; Voigt et al., 2014	↑ Shanmugam et al., 2011	↓ Yeh et al., 2008; Johnsen et al., 2013; Clarke et al., 2015; Hohendanner et al., 2016
				↔ Kneller et al., 2002	
				↓ Voigt et al., 2012	
	*PLB mRNA*	–	–	↔ Lai et al., 1999; Van Gelder et al., 1999	–
				↓ Gaborit et al., 2005	
	*PLB protein expression*	↔ Greiser et al., 2014	↔ Brundel et al., 1999; Voigt et al., 2014	↔ Brundel et al., 1999; Schotten et al., 2002; Uemura et al., 2004; El-Armouche et al., 2006; Lenaerts et al., 2009; Neef et al., 2010	↔ Yeh et al., 2008; Shanmugam et al., 2011; Clarke et al., 2015
				↓ Hoit et al., 2002	
	*PLB phosphorylation (Ser16)*	↔ Greiser et al., 2014	↓ Greiser et al., 2009; Lugenbiel et al., 2015 ↑ Voigt et al., 2014	↑ El-Armouche et al., 2006; Dai et al., 2016 ↔ Lenaerts et al., 2009	↓ Shanmugam et al., 2011
	*PLB phosphorylation (Thr17)*	↔ Greiser et al., 2014	↔ Voigt et al., 2014 ↑ Chelu et al., 2009	↑ El-Armouche et al., 2006	↑ Yeh et al., 2008; Clarke et al., 2015
			↓ Lugenbiel et al., 2015	↔ Neef et al., 2010	
	*Sarcolipin mRNA*	–	–	↓ Uemura et al., 2004	–
	*Sarcolipin protein expression*	–	↓ Xie et al., 2012	↓ Shanmugam et al., 2011	↓ Shanmugam et al., 2011
RyR	*RyR mRNA*	–	↔ Brundel et al., 1999	↔ Brundel et al., 1999; Lai et al., 1999	–
				↓ Ohkusa et al., 1999	
	*RyR protein expression*	↓ Greiser et al., 2014	↑ Voigt et al., 2014	↓ Ohkusa et al., 1999; Lenaerts et al., 2009; Neef et al., 2010	↔ Yeh et al., 2008; Shanmugam et al., 2011
			↔ Brundel et al., 1999; Wakili et al., 2010	↔ Brundel et al., 1999; Schotten et al., 2002; Voigt et al., 2012	↓ Brandenburg et al., 2016
				↑ Dai et al., 2016	
	*RyR phosphorylation (Ser2808)*	↑ Greiser et al., 2014	↔ Chelu et al., 2009; Wakili et al., 2010; Chiang et al., 2014b; Voigt et al., 2014	↑ Vest et al., 2005; Voigt et al., 2012	↑ Brandenburg et al., 2016
			↑ Li et al., 2014		
			↓ Lugenbiel et al., 2015		↓ Yeh et al., 2008
	*RyR phosphorylation (Ser2814)*	↓ Greiser et al., 2014	↑ Chelu et al., 2009; Greiser et al., 2009; Chiang et al., 2014b	↑ Neef et al., 2010; Voigt et al., 2012	↔ Yeh et al., 2008; Brandenburg et al., 2016
			↔ Wakili et al., 2010; Voigt et al., 2014; Lugenbiel et al., 2015		
	*Single channel open probability*	–	↑ Beavers et al., 2013; Chiang et al., 2014b; Voigt et al., 2014	↑ Vest et al., 2005; Neef et al., 2010; Voigt et al., 2012; Macquaide et al., 2015	–
				↔ Lenaerts et al., 2009	
NCX	*NCX mRNA*	–	↔ Brundel et al., 1999	↔ Brundel et al., 1999; Van Gelder et al., 1999; Yue et al., 1999; Uemura et al., 2004	–
	*NCX protein expression*	↔ Greiser et al., 2014	↔ Brundel et al., 1999; Wakili et al., 2010; Voigt et al., 2014	↑ Schotten et al., 2002; El-Armouche et al., 2006; Lenaerts et al., 2009; Voigt et al., 2012	↑ Li et al., 2000
			↑ Lugenbiel et al., 2015	↔ Brundel et al., 1999; Hoit et al., 2002	
	*I_*ti*_*	↑ Greiser et al., 2014	↔ Cha et al., 2004a; Wakili et al., 2010; Voigt et al., 2014	↑ Lenaerts et al., 2009; Voigt et al., 2012	↑ Li et al., 2000; Cha et al., 2004a,b; Johnsen et al., 2013; Hohendanner et al., 2015, 2016
				↔ Kneller et al., 2002	↓ Clarke et al., 2015
Calcium Transient Amplitude		↓ Sun et al., 1998; Greiser et al., 2014	↔ Voigt et al., 2014	↓ Schotten et al., 2002; Lenaerts et al., 2009; Voigt et al., 2012	↓ Saba et al., 2005; Bonilla et al., 2014; Clarke et al., 2015
		↔ Wakili et al., 2010		↑ Yeh et al., 2008; Hohendanner et al., 2016
Afterdepolarizations	*Spark and wave frequency*	↔ Greiser et al., 2014	↑ Hove-Madsen et al., 2004; Sood et al., 2008; Chelu et al., 2009; Li N. et al., 2012; Shan et al., 2012; Beavers et al., 2013; Chiang et al., 2014a,b; Faggioni et al., 2014; Li et al., 2014; Voigt et al., 2014	↑ Neef et al., 2010; Voigt et al., 2012; Purohit et al., 2013; Macquaide et al., 2015	↑ Saba et al., 2005; Yeh et al., 2008; Johnsen et al., 2013; Hohendanner et al., 2015, 2016; Aistrup et al., 2017
	*EADs*	–	↑ Burashnikov and Antzelevitch, 2003	–	–
	*DADs*	–	↑ Li N. et al., 2012; Chiang et al., 2014a; Faggioni et al., 2014; Li et al., 2014; Voigt et al., 2014	↑ Voigt et al., 2012; Purohit et al., 2013	Yeh et al., 2008; Hohendanner et al., 2015, 2016; Aistrup et al., 2017

*Early = Animal models with <7 days atrial tachycardia pacing. pAF (paroxysmal atrial fibrillation) = Animal models with 7–28 days of atrial tachycardia pacing OR a genetically modified mouse model of AF OR a human study detailing paroxysmal AF in the baseline characteristics. PerAF (persistent atrial fibrillation) = Animal models with >28 days of atrial tachycardia pacing OR human studies with either: persistent AF reported in baseline characteristics or assumed persistent in absence of description*.

Structurally, the loss or remodeling of t-tubules results in RyRs becoming “orphaned” and producing dyssyncronous calcium sparks which may promote calcium dependant arrhythmias in the ventricle (Song et al., 2006). However the role of orphaned ventricular RyRs is unclear since in dyssyncronous heart failure loss of t-tubules from the cell ends is associated with higher RyR density and a decrease in calcium sparks (Li et al., 2015). While orphaned RyRs will inevitably arise following atrial t-tubule loss, whether they promote atrial arrhythmias has not yet been shown. It is well known that t-tubule loss reduces the synchrony of ventricular calcium release (Louch et al., 2006; Heinzel et al., 2008) which is aggravated by failed action potential propagation into remodeled t-tubules (Sacconi et al., 2012). Similarly, in the atria, t-tubule loss is associated with impaired calcium release in the cell interior (Dibb et al., 2009; Lenaerts et al., 2009; Wakili et al., 2010). Both orphaned RyRs and heterogeneous calcium release have been suggested to contribute to the increased alternans susceptibility which computer models predict to occur due to t-tubule disruption and loss (Li Q. et al., 2012; Nivala et al., 2015) providing a mechanism by which t-tubule associated changes in calcium handling may facilitate AF.

### How do the atria respond to excess calcium influx acutely?

Rapid atrial stimulation by triggering impulses increases cellular calcium loading in the atria (Sun et al., 2001). It might be expected that the heart would have a mechanism to protect itself against rapid stimulation that would otherwise cause uncontrolled calcium overload, and this could be achieved by reducing calcium influx or increasing calcium efflux. To explore whether such a mechanism does indeed exist, animal models of short-term rapid atrial pacing have been used. One adaptive mechanism that has recently come to light is calcium signal silencing (Greiser et al., 2014) which affects both calcium influx and efflux.

Calcium influx is primarily determined by *I*_Ca(L)_ which is decreased in the early stages of rapid atrial pacing both in terms of current density and LTCC expression (Table 1). Studies investigating rapid pacing (up to 7 days duration) show a progressive reduction in *I*_Ca(L)_ (Bosch et al., 2003; Qi et al., 2008) with decreased mRNA encoding the alpha subunit of the LTCC seen after just 24 h (Bosch et al., 2003). The downregulation in mRNA transcription is believed to be mediated by the acute increase in intracellular calcium acting via the calcium calmodulin-calcineurin-NFAT pathway to help keep cells in calcium balance (Qi et al., 2008).

Calcium efflux is also increased in these early stages of tachypacing. This occurs via an increase in NCX current for a given level of intracellular calcium as opposed to any change in NCX expression (Greiser et al., 2014). Taken alone this might be expected to increase both action potential duration and DADs via increased *I*_ti_, depolarizing the sarcolemma reaching the threshold for triggering additional action potentials, however, neither of these occur. Signal silencing firstly involves shortening of the action potential where presumably factors which include decreased *I*_Ca(L)_ offset any increase in I_NCX_. Secondly, the rate of arrhythmogenic calcium release in the form of sparks is unaltered since increased I_NCX_ is balanced by protective changes such as decreased RyR expression and reduced phosphorylation by calcium calmodulin dependent kinase two (CaMKII), decreased intracellular sodium, and increasing intracellular calcium buffering (Greiser, 2017). Overall these factors in combination with a lack of change in SR calcium content are the hallmarks of silencing that result in no observable change in calcium sparks and a reduction in calcium transient amplitude (Sun et al., 2001; Greiser et al., 2014).

Calcium signal silencing has, to the best of our knowledge, only been studied in a species where atrial t-tubules are generally absent (Greiser et al., 2014). It is therefore unknown if early t-tubule loss, as a result of rapid atrial stimulation (Wakili et al., 2010), could facilitate signal silencing by reducing central calcium release or if early t-tubule loss promotes calcium dependent arrhythmias favoring the progression from signal silencing to AF.

### Which aspects of calcium cycling promote paroxysmal atrial fibrillation?

Paroxysmal AF, characterized by initially short periods of self-terminating AF, can be found when the triggers for AF are present but the atrial substrate has not yet remodeled to an extent that supports persistent AF. Remodeling of calcium cycling is an important step in the temporal progression to paroxysmal AF. This will involve additional changes in calcium handling not seen in silencing which ultimately lead to arrhythmogenic calcium release and triggered activity thereby promoting AF.

#### Paroxysmal atrial fibrillation is associated with diastolic leak from ryanodine receptors

A key concept in the pathophysiology of paroxysmal AF is increased diastolic leak of calcium from the SR, which can be quantified by assessing the frequency of calcium sparks and arrhythmogenic calcium waves. An increase in calcium sparks and waves is well known to occur in paroxysmal AF (Table 1) and is associated with RyR remodeling in a manner that favors calcium release. Whereas in the early stages of rapid atrial stimulation that lead to signal silencing RyR expression was decreased, in paroxysmal AF the trend is toward an increase in expression (Brundel et al., 1999; Greiser et al., 2014; Voigt et al., 2014) with an increase in channel open probability (Beavers et al., 2013; Chiang et al., 2014b; Voigt et al., 2014). In these studies the main driver was thought to be an increase in RyR phosphorylation. RyRs can be phosphorylated by both PKA (at Ser2808) and CaMKII (at Ser2814). In paroxysmal AF increased RyR open probability appears to arise from phosphorylation at the CaMKII site whereas results regarding phosphorylation at the PKA site are mixed (Chelu et al., 2009; Wakili et al., 2010; Chiang et al., 2014b; Li et al., 2014; Voigt et al., 2014; Lugenbiel et al., 2015). In terms of the CaMKII site evidence suggests a progression from the earliest effects of signal silencing in which RyR phosphorylation decreases (Greiser et al., 2014), to the majority of papers in paroxysmal AF reporting an increase in RyR phosphorylation (Chelu et al., 2009; Greiser et al., 2009; Chiang et al., 2014b). For sustained calcium leak to occur a mechanism is required to maintain SR calcium loading and this is discussed in the following section. It is unknown if t-tubule loss (Wakili et al., 2010), orphaned RyRs or RyR remodeling (Song et al., 2006; Li et al., 2015) play a role in increased calcium sparks and waves in paroxysmal AF. Following myocardial infarction in the ventricle, CaMKII modulates only non-coupled RyRs (Dries et al., 2013) but whether this mechanism promotes calcium leak in the atria at sites following t-tubule loss remains unknown.

Diastolic leak may also be implicated in rare inherited forms of paroxysmal AF. Expressing mutations found in families with inherited AF syndromes in mouse models has found that these variants are frequently associated with RyR remodeling. Knock-out of spinophilin-1 (a protein that links RyRs to protein phosphatase (1) in mice leads to hyperphosphorylation of RyRs at the CaMKII site and an increase in leak (Chiang et al., 2014b). Mice with a loss of function mutation in junctophilin (a protein which normally binds and stabilizes RyRs) show reduced junctophilin binding to RyR, with a secondary increase in open probability and leak (Beavers et al., 2013). Knock-out mice for FKBP-12.6 (a protein which binds and stabilizes RyRs in their unphosphorylated state) show increased leak (Sood et al., 2008), which can be reversed by inhibiting CaMKII phosphorylation of RyR (Li N. et al., 2012). Finally, a genetic mutation resulting in loss of miRNA-106b-25 cluster increases vulnerability to AF by increasing RyR expression (Chiang et al., 2014a). Overall, increased leak in paroxysmal AF appears to be via CaMKII phosphorylation of RyRs. As paroxysms of AF continue to increase cellular calcium concentrations, and as CaMKII activity is regulated by the concentration of calcium, it is easy to imagine how this could result in a positive feedback loop perpetuating AF.

#### How is RyR leak maintained and translated to triggered activity in paroxysmal atrial fibrillation?

In addition to the properties of RyRs, diastolic leak is also dependent on SR calcium content, with higher SR calcium content leading to greater diastolic leak (Lukyanenko et al., 1996). Importantly, simply making the RyR leaky without otherwise manipulating SR content will not produce calcium waves in the steady state—while this decreases the threshold for a calcium wave, it also decreases SR calcium content below the threshold (Diaz et al., 1997; Venetucci et al., 2007). Studies investigating the absolute SR content in paroxysmal AF are inconsistent (Hove-Madsen et al., 2004; Greiser et al., 2009; Wakili et al., 2010; Voigt et al., 2014) and the relationship to threshold is unknown. However, all studies we refer to in (Table 1) report an increase in diastolic calcium leak in paroxysmal AF therefore we assume SR calcium content must be above threshold.

For calcium waves to occur a mechanism is required to maintain SR calcium content above threshold (Venetucci et al., 2007; Ho et al., 2016). SERCA function increases in paroxysmal AF (Xie et al., 2012; Voigt et al., 2014) which would explain how SR calcium content could be maintained in spite of an increase in RyR mediated calcium leak. The increase in SERCA function occurs despite decreased expression of SERCA protein (Voigt et al., 2014; Lugenbiel et al., 2015; Wang et al., 2017), potentially caused by changes in regulatory proteins. Phospholamban (PLB) inhibits SERCA function although this inhibition can be relieved by phosphorylation by PKA (Ser16 site) or CaMKII (Thr17 site). PLB protein expression does not change in paroxysmal AF and investigation of its phosphorylation status has produced inconsistent results (Brundel et al., 1999; Chelu et al., 2009; Greiser et al., 2009; Voigt et al., 2014; Lugenbiel et al., 2015). In the atria, in addition to PLB, sarcolipin can also slow calcium reuptake by SERCA. We are unaware of any studies measuring sarcolipin levels in paroxysmal AF. However, sarcolipin knock-out mice show increased SERCA function and an increased frequency of afterdepolarizations, supporting the concept of maintained SR calcium content to promote leak (Babu et al., 2007; Xie et al., 2012). It is important to appreciate that increasing SERCA function can have either pro- or anti-arrhythmic effects in different contexts. While increasing SERCA function could overload the SR with calcium and thereby promote afterdepolarizations, increased SERCA function might also be expected to increased calcium buffering and thereby increase the threshold for calcium waves (Briston et al., 2014). Reflecting these different possibilities, while increasing SERCA by gene transfer in the failing ventricle is anti-arrhythmic (Lyon et al., 2011), overexpressing SERCA in mouse atria promotes cellular correlates of AF (Nassal et al., 2015).

NCX is important in the conversion of calcium leak to arrhythmias as the calcium released by a wave is removed from the cytosol by NCX to generate the transient inward current *I*_ti_ which can trigger additional action potentials (Ferrier et al., 1973). The increase in calcium sparks and waves in paroxysmal AF is associated with an increase in afterdepolarizations (Li N. et al., 2012; Chiang et al., 2014a; Faggioni et al., 2014; Li et al., 2014; Voigt et al., 2014). NCX remains relatively unchanged in paroxysmal AF compared to sinus rhythm (Table 1), although as NCX increased in the early stages of rapid atrial stimulation that led to calcium signal silencing, the normalization of NCX in paroxysmal AF may represent a fall from an initial rise. However, insufficient evidence exists at this time to form firm conclusions particularly around what drives the initial rise and subsequent fall in NCX function as this does not relate to expression levels (Table 1).

Similar to the acute response to rapid atrial pacing, both the current density of *I*_Ca(L)_ and the expression of LTCCs are reduced in paroxysmal AF (Brundel et al., 2001; Yagi et al., 2002; Cha et al., 2004a; Wakili et al., 2010; Lugenbiel et al., 2015). It is important to recognize that although *I*_Ca(L)_ density is generally decreased, consistent with protection against calcium overload, this is offset by rapid atrial rates during paroxysms of AF which would be expected to promote calcium loading. Additionally decreased *I*_Ca(L)_ could have further pro-arrhythmic effects due to a shortening of the action potential duration, decreasing the refractory period and therefore the wavelength of potential re-entrant circuits.

Overall, paroxysmal AF is associated with increased RyR diastolic leak, increased SERCA function despite unchanged SERCA expression, decreased *I*_Ca(L)_ and t-tubule loss. These changes appear to initiate a stepwise progression of remodeling toward persistent AF in which an atrial substrate develops which can support increasing durations of AF. This remodeling includes a positive feedback mechanism in which activation of CaMKII phosphorylates RyRs increasing diastolic leak and encouraging further rises in intracellular calcium (Qi et al., 2008), and substrate development by upregulation of pro-fibrotic pathways such as the calcium calmodulin-calcineurin-NFAT pathway (Lin et al., 2004; Wakili et al., 2011). A major question remains namely by what mechanism does early silencing progress to paroxysmal AF or, alternatively, why do some patients develop AF and others not? One possible explanation may lie in the ability of the heart to prevent calcium overload. Interestingly, when considering susceptibility to AF, it was the patients with the greatest *I*_Ca(L)_ who had an increased incidence of post-operative AF (Van Wagoner et al., 1999). We might speculate that a lack of ability to adapt to high rate by decreasing *I*_Ca(L)_ and protecting the atria from excessive calcium loading could potentiate the development of paroxysms of AF via calcium dependant arrhythmias.

### How does remodeling of calcium cycling allow paroxysmal atrial fibrillation to progress to persistent atrial fibrillation?

The duration of the paroxysms of AF tends to prolong until persistent AF develops, defined clinically as episodes of arrhythmia that last more than seven days before returning to sinus rhythm spontaneously, if at all (Kirchhof et al., 2016). The progression to persistent AF involves alterations in the atrial substrate and calcium handling such that ongoing elevations in cytosolic calcium concentrations activate pathways leading to structural remodeling of the atria. This, coupled with the continued decrease in refractory period leads to the atrial substrate becoming more vulnerable and assuming a greater role in the maintenance of the arrhythmia (Cha et al., 2004b).

#### SR calcium leak in persistent atrial fibrillation is associated with RyR phosphorylation at both PKA and CaMKII sites

The enhanced diastolic leak of calcium found in paroxysmal AF continues as AF becomes persistent, and continues to promote triggered activity (Voigt et al., 2012; Purohit et al., 2013). RyR expression tends to be unaltered or downregulated (Brundel et al., 1999; Ohkusa et al., 1999; Schotten et al., 2002; Lenaerts et al., 2009; Neef et al., 2010; Voigt et al., 2012) and is unlikely to be a major factor in the increased SR calcium leak observed in persistent AF. Instead, leak may be due altered kinetics of RyR opening. As was seen in paroxysmal AF, RyR single channel open probability is increased in persistent AF (Vest et al., 2005; Neef et al., 2010; Voigt et al., 2012; Macquaide et al., 2015), which may be due to RyR phosphorylation.

Persistent AF is associated with increased phosphorylation at both the PKA and CaMKII site as well as hyperphosphorylation at the CaMKII site (Vest et al., 2005; Neef et al., 2010; Voigt et al., 2012). Hyperphosphorylation is thought to arise when all four RyR subunits are phosphorylated instead of just two (Marx et al., 2000; Voigt et al., 2012) further increasing their diastolic open probability and leading to a higher frequency of calcium sparks, waves and afterdepolarizations (Vest et al., 2005; Voigt et al., 2012). This is in contrast to paroxysmal AF where RyR leak appeared to be predominantly via CaMKII phosphorylation (Table 1).

Experiments blocking RyR phosphorylation suggest that the CaMKII site may be more important than the PKA site in determining SR calcium leak in persistent AF (Neef et al., 2010; Voigt et al., 2012), although the relative role of PKA vs. CaMKII in promoting SR calcium leak is controversial. Evidence of the importance of RyR phosphorylation by PKA come from studies suggesting that PKA phosphorylation leads to dissociation of FKBP12.6 from the RyR, enhancing the open probability of the channel and facilitating SR calcium leak and arrhythmias (Marx et al., 2000; Lehnart et al., 2006). However, this is not universally accepted as other laboratories suggest that hyperphosphorylation of RyR by PKA is not involved in cardiac dysfunction (Benkusky et al., 2007; Zhang et al., 2012). For detailed reviews on this topic we refer the reader to Houser (2014), Dobrev and Wehrens (2014) or Landstrom et al. (2017).

#### The mechanisms maintaining SR calcium content despite increased leak in persistent atrial fibrillation are unclear

As was seen in paroxysmal AF, maintaining SR calcium leak requires a mechanism to maintain SR calcium content. Despite the increase in diastolic leak, SR calcium content does not change in persistent AF (Lenaerts et al., 2009; Neef et al., 2010; Voigt et al., 2012). While in paroxysmal AF this is likely to be due to increased SERCA function, the mechanism responsible for maintaining SR calcium content in persistent AF is unclear. In persistent AF, SERCA protein and mRNA levels are generally similar to control (Hoit et al., 2002; Schotten et al., 2002; El-Armouche et al., 2006; Lenaerts et al., 2009; Neef et al., 2010; Dai et al., 2016) or may even decrease (Brundel et al., 1999; Lai et al., 1999; Ohkusa et al., 1999; Cao et al., 2002) While expression of PLB does not appear to change (Brundel et al., 1999; Lai et al., 1999; Van Gelder et al., 1999; Schotten et al., 2002; Uemura et al., 2004; El-Armouche et al., 2006; Lenaerts et al., 2009; Neef et al., 2010), phosphorylation of PLB may increase (El-Armouche et al., 2006; Dai et al., 2016) but this is not universally reported (Lenaerts et al., 2009; Neef et al., 2010). It is also unclear why differential CaMKII phosphorylation of PLB and RyR can occur within the same atria (Neef et al., 2010) raising the possibility that CaMKII signaling is compartmentalized (Mishra et al., 2011). It has also been shown that sarcolipin expression is reduced in persistant AF, providing an additional mechanism to increase SERCA function (Uemura et al., 2004; Shanmugam et al., 2011). However, inconsistent results have been reported when SERCA function has been directly measured in persistent AF (Kneller et al., 2002; Shanmugam et al., 2011; Voigt et al., 2012).

#### How does the structure and function of the sarcolemma remodel in persistant atrial fibrillation?

NCX expression and current may increase in response to high burdens of persistent AF although these findings are not universal (Brundel et al., 1999; Hoit et al., 2002; Kneller et al., 2002; Schotten et al., 2002; El-Armouche et al., 2006; Lenaerts et al., 2009; Voigt et al., 2012). A trend toward an increase in *I*_ti_ would support the hypothesis that an increase in diastolic leak may result in the increased occurrence of afterdepolarizations that might reinitiate or sustain persistent AF (Burashnikov and Antzelevitch, 2003, 2006). However NCX expression is not associated with the duration of AF in man (Brundel et al., 1999; Van Gelder et al., 1999).

The decrease in *I*_Ca(L)_ seen early in response to rapid atrial stimulation continues in response to repeated episodes of AF (Yue et al., 1997; Van Wagoner et al., 1999; Workman et al., 2001; Yagi et al., 2002; Christ et al., 2004; Lenaerts et al., 2009; Voigt et al., 2012), potentially caused by lower levels of mRNA for the alpha subunit (Brundel et al., 1999, 2001; Lai et al., 1999; Van Gelder et al., 1999; van der Velden et al., 2000b; Gaborit et al., 2005), reduced LTCC protein expression (Brundel et al., 1999, 2001; Lenaerts et al., 2009), a shift in single channel gating (Lenaerts et al., 2009) and potentially a reduction in single channel open probability due to reduced phosphorylation (Christ et al., 2004). The time course over which these changes occur has been variably reported as between 2 and 6 months (Van Gelder et al., 1999; van der Velden et al., 2000b), which may reflect differences in AF burden between studies. Studies investigating LTCCs during the progression of AF report decreased expression in persistent AF but not in paroxysmal AF suggesting *I*_Ca(L)_ might decrease over the time course of the disease (Brundel et al., 1999; Van Gelder et al., 1999). However others report no change in *I*_Ca(L)_ between paroxysmal and persistent AF (Yagi et al., 2002). This discrepancy between decreased expression and current density might be in part due to the reduction in calcium transient amplitude in persistent AF (Schotten et al., 2002; Lenaerts et al., 2009; Voigt et al., 2012) resulting in decreased calcium dependent inactivation of *I*_Ca(L)_ maintaining current amplitude. These differences may also explain why some studies report the nadir of *I*_Ca(L)_ is reached quickly before remaining stable, while others reported a slow downward trend inversely proportional to increasing amounts of time in AF (Yagi et al., 2002; Cha et al., 2004a; Wakili et al., 2010).

While atrial t-tubule loss contributes to the decrease of *I*_Ca(L)_ in AF in some species (Lenaerts et al., 2009), the absence of atrial t-tubules at baseline in other species precludes the involvement of this mechanism e.g., Greiser et al. (2014) and likely contributes to disparity between studies regarding the time course of *I*_Ca(L)_ loss. While t-tubule loss could increase as AF develops and contribute to the progressive loss of *I*_Ca(L)_ the available data does not support this concept. Following 7 days of rapid atrial pacing in the dog t-tubules were reduced by 60% (Wakili et al., 2010) but only by ~45% following 182 days of rapid atrial pacing in the sheep (Lenaerts et al., 2009) suggesting t-tubule loss may facilitate AF in the early stages but not the subsequent progression of AF.

Electrically, the decrease in *I*_Ca(L)_ contributes to the progressive shortening of action potential duration seen in persistent AF (Bosch et al., 1999; Workman et al., 2001; Kneller et al., 2002; Wakili et al., 2010; Schmidt et al., 2015). This shortening decreases the refractory period of the atrial myocytes, in turn reducing the wavelength of potential re-entrant circuits, allowing more circuits to co-exist within a given mass of atrial tissue and reducing the likelihood of AF terminating. The refractory period not only decreases, but also loses the ability to adapt to changes in heart rate (Wijffels et al., 1995; Gaspo et al., 1997b; Bosch et al., 1999; Willems et al., 2001; Workman et al., 2001; Cha et al., 2004a; Todd et al., 2004; Anne et al., 2007). These changes in action potential duration and refractory period are also contributed to by a reduction in *I*_Na_ (Gaspo et al., 1997a) and *I*_to_ (Le Grand et al., 1994; Yue et al., 1997; Bosch et al., 2003) and an increase in *I*_K1_(Workman et al., 2001; Dobrev et al., 2005). A full review of all ion channel remodeling at the various stages of AF is beyond the scope of this article and the reader is directed to Nattel et al. (2007).

#### Elevated cytosolic calcium activates pro-fibrotic pathways leading to structural remodeling

Calcium also plays a key role in the structural changes in the atria caused by and facilitating the progression of persistent AF. Despite the efforts of the myocyte to minimize calcium entry via *I*_Ca(L)_, if calcium silencing fails then cytosolic calcium concentrations inevitably rise. This rise activates intracellular signaling pathways such as the pro-fibrotic Wnt pathway via calcium sensitive enzymes such as protein kinase C, CaMKII and calcineurin, and leads to myocyte hypertrophy, fibrosis and atrial dilatation (Bukowska et al., 2006; De, 2011; Tao et al., 2016). The consequence of atrial dilatation is a greater mass of tissue able to harbor more simultaneous re-entrant circuits, stabilizing the arrhythmia. In response to increased oxidative stress and angiotensin-II levels in AF (Tsai et al., 2011), pro-fibrotic pathways including the fibroblast transforming growth factor beta pathway (Harada et al., 2012) encourage expansion of the extracellular matrix with deposition of fibrillin and fibronectin, providing a substrate for re-entry by increasing heterogeneity of impulse conduction (Spach et al., 1982; Li et al., 1999). Furthermore, the gap junctions that electrically connect adjacent myocytes are remodeled by AF, altering the relative expression of their constituent proteins connexins 40, 43, and 45 and further disturbing atrial conduction (Elvan et al., 1997; van der Velden, et al., 2000a; Dupont et al., 2001). Atrial structural remodeling may be facilitated in part by changes in calcium cycling within fibroblasts. For reviews of the role of calcium in dictating structural remodeling in atrial myocytes and fibroblasts we refer the interested reader to reviews by Wakili et al. (2011) and Nattel (2017).

#### Elevated intracellular calcium promotes mitochondrial dysfunction

An additional effect of increasing intracellular calcium concentrations is disturbed mitochondrial function. Calcium cycling is an energetic process which is dependent upon the bidirectional relationship between the SR and the mitochondria (as reviewed in Dorn and Maack, 2013). Mitochondria act as a calcium buffer, and the increased cytosolic calcium also leads to increased calcium concentrations within the mitochondria (Ausma et al., 2000). This increases the open probability of the calcium-sensitive mitochondrial permeability transition pore resulting in increased proton leak and disrupting ATP synthesis (Parks et al., 2018). The proton leak in turn generates reactive oxygen species (ROS) and promotes cell death (reviewed in Griffiths, 2012). The mitochondrial dysfunction that can occur at times of calcium overload is compounded by the increased energy demand of active transporters such as SERCA which are trying to restore homeostatic balance. If these energy demands are not met, mitochondrial dysfunction can contribute to calcium cycling remodeling including increasing RyR leak (Anzai et al., 1998; Xie et al., 2015) and a greater frequency of DADs (Beresewicz and Horackova, 1991).

The effects of mitochondrial dysfunction, increased ROS production and increased oxidative stress have been observed in patients with persistent AF (Mihm et al., 2001; Bukowska et al., 2008; Yongjun et al., 2013; Xie et al., 2015) and can also can predict vulnerability to AF following cardiac surgery (Carnes et al., 2001; Montaigne et al., 2013; Anderson et al., 2014). A full review of ROS and oxidative stress in AF can be found in Sovari et al. (Sovari and Dudley, 2012).

#### Summary of the role of calcium handling in atrial fibrillation

In summary, triggering impulses, arising most commonly from the pulmonary veins, lead to short bursts of rapid atrial activity and an increase in the influx of calcium. The response of the healthy atrium is to offset this net influx by increasing efflux in calcium signal silencing. However since some patients develop AF there must be factors which promote paroxysmal AF rather than signal silencing. We speculate that this could include (i) a failure in the ability of the calcium cycling mechanism to adapt to increased rate, (ii) the presence of pre-existing structural heart disease or, (iii) early loss of atrial t-tubules promoting arrhythmogenic calcium release. Other, extra cardiac factors, may also also determine the vulnerability to AF and include roles for obesity (Goudis et al., 2015), aging (Steenman and Lande, 2017) or alcohol (Voskoboinik et al., 2016), which could further promote arrhythmogenic calcium release. These factors, either individually or in combination may determine the probability of developing persistent AF as well as the rate of progression of the disease. It is clear that changes to calcium cycling are a key component of this progression encouraging electrical remodeling, structural remodeling, and mitochondrial dysfunction, and ultimately promoting further AF. A downward spiral ensues until the atrium is capable of sustaining AF indefinitely, making it harder to restore and sustain sinus rhythm.

## The interplay between heart failure and atrial fibrillation

**Figure 3:**
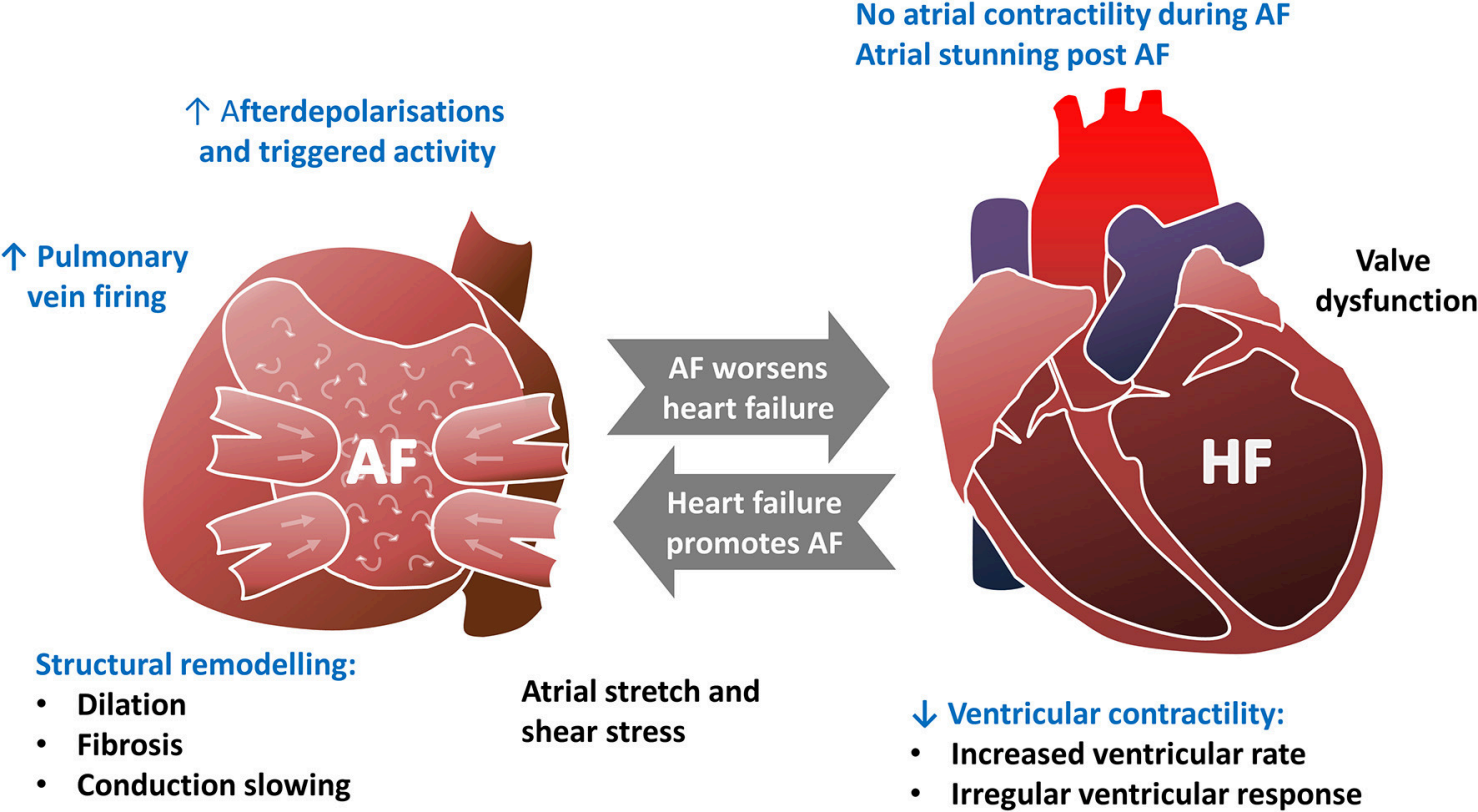
The bidirectional relationship between atrial fibrillation and heart failure. Text highlighted in Blue indicates that perturbed calcium handling is implicated in this aspect of the pathophysiology.

Heart failure is a leading cause of death worldwide that arises as an endpoint of many cardiovascular diseases including ischaemic heart disease, valvular heart disease, hypertension, and inherited cardiomyopathies (Ziaeian and Fonarow, 2016). In addition to ventricular arrhythmias that can lead to sudden cardiac death (Bardy et al., 2005), heart failure is also associated with an increased risk of atrial arrhythmias including AF (Benjamin et al., 1994). AF, if seen, is associated with a worsening of symptoms and prognosis (Dries et al., 1998; McManus et al., 2013; Odutayo et al., 2017). The relationship between heart failure and AF is summarized in Figure 3.

Heart failure is defined as the presence of symptoms such as breathlessness resulting from structural or functional abnormalities that in general cause impaired contraction and / or relaxation of the myocardium (Ponikowski et al., 2016). This broad definition aids clinicians in adopting a standard approach to patient investigation and treatment, but generates a heterogeneous group that requires further subdivision in order to make sense of the range of human and animal studies performed. The commonest way of subdividing heart failure based on cardiac performance is into two groups: heart failure with a reduced ejection fraction vs. heart failure with a preserved ejection fraction (Ponikowski et al., 2016). The majority of studies examining calcium handling in the atria in heart failure have focused on those with reduced ejection fraction and we will therefore focus on this group, although it is important to remember that all types of heart failure are associated with AF (McManus et al., 2013).

In this section, we will not only show that the atria in heart failure undergo remodeling in calcium cycling, but this bears a number of similarities to the remodeling in AF presented in the previous section and that the two conditions are inexorably linked.

### Does heart failure promote atrial fibrillation through changes in atrial calcium cycling?

In contrast to the large body of work exploring the role of calcium handling in AF, far less has been published regarding the effects of heart failure on atrial calcium handling. However, heart failure appears to be associated with changes to atrial calcium cycling with decreased *I*_Ca(L)_ contributed to by loss of atrial t-tubules, increased SR calcium content despite unchanged or lower calcium reuptake by SERCA, and increased diastolic calcium leak. These effects may be mediated by atrial stretch caused by elevated intra-atrial pressure (Eckstein et al., 2008). Heart failure also affects the pulmonary veins, increasing their spontaneous activity (Lin et al., 2016). Overall many aspects of calcium handling remodeling are common to both AF and heart failure and therefore it is plausible that AF could exacerbate heart failure remodeling and vice versa.

#### Heart failure is associated with decreased atrial I_Ca(L)_ and increased sarcoplasmic reticulum calcium content

Atrial *I*_Ca(L)_ has generally been found to decrease in heart failure (Ouadid et al., 1995; Li et al., 2000; Boixel et al., 2001; Cha et al., 2004a,b; Sridhar et al., 2009; Clarke et al., 2015) although these findings have not been universally replicated in human studies (Cheng T. H. et al., 1996; Workman et al., 2009). Interestingly, this reduction in *I*_Ca(L)_ has also been found in those with dilated atria and other forms of structural heart disease associated with an increased susceptibility to AF (Le Grand et al., 1994; Deroubaix et al., 2004; Dinanian et al., 2008). The reduction in *I*_Ca(L)_ appears to be due to a reduction in the expression of LTCCs (Ouadid et al., 1995). Decreased atrial t-tubule density may contribute to the reduced abundance of LTCCs (Dibb et al., 2009; Lenaerts et al., 2009). T-tubules are disordered in hypertrophied atria (Brandenburg et al., 2016) and lost in heart failure and myocardial infarction (Dibb et al., 2009; Kettlewell et al., 2013; Caldwell et al., 2014) to a greater extent than is seen in the ventricle (Dibb et al., 2009; Caldwell et al., 2014). In addition the amplitude of *I*_Ca(L)_ on the t-tubules that remain in heart failure is also reduced (Glukhov et al., 2015) further decreasing overall *I*_Ca(L)._ The decrease in atrial *I*_Ca(L)_ is contributed to by a reduced baseline response to beta stimulation (Boixel et al., 2001), while single channel voltage gating and recovery from inactivation do not appear to change (Li et al., 2000; Boixel et al., 2001; Cha et al., 2004b). Overall, there appears to be a decrease in atrial *I*_Ca(L)_ in heart failure (Ouadid et al., 1995; Li et al., 2000; Boixel et al., 2001; Cha et al., 2004a,b,b; Dinanian et al., 2008; Sridhar et al., 2009; Clarke et al., 2015) which can also have effects on SR calcium content (Trafford et al., 2001; Clarke et al., 2015, 2017).

Increasing SR calcium content is generally pro-arrhythmic as this can lead to an increased frequency of delayed afterdepolarizations, and this is often seen in the atria of those with heart failure (Yeh et al., 2008; Hohendanner et al., 2015, 2016; Aistrup et al., 2017). While in rodents a decrease in SR calcium content (Saba et al., 2005) is associated with reduced SERCA, PLB, and sarcolipin expression (Shanmugam et al., 2011), a somewhat different story predominates in large mammals in which atrial SR calcium content increases in heart failure (Yeh et al., 2008; Clarke et al., 2015; Aistrup et al., 2017) despite decreased calcium reuptake by SERCA (Clarke et al., 2015; Hohendanner et al., 2016). This apparently paradoxical finding has been explained by the decrease in atrial *I*_Ca(L)_ where inhibiting *I*_Ca(L)_ in control cells reproduces the increase in SR calcium content. Here, decreased *I*_Ca(L)_ can paradoxically increase SR calcium content (Trafford et al., 2001; Clarke et al., 2015, 2017). It is less clear how SERCA function is reduced in these models of heart failure. Generally in large mammals and man, SERCA and PLB levels remain unchanged but can decrease (Yeh et al., 2008; Shanmugam et al., 2011; Clarke et al., 2015). However, other changes have been observed which would be expected to increase SERCA function in heart failure including an increase in phosphorylation of PLB at the CaMKII site (Yeh et al., 2008; Clarke et al., 2015) and a decrease in sarcolipin levels (Shanmugam et al., 2011).

In keeping with the recurring theme seen in paroxysmal and persistent AF, heart failure is also associated with increased diastolic calcium leak (Saba et al., 2005; Yeh et al., 2008; Johnsen et al., 2013; Hohendanner et al., 2015, 2016; Aistrup et al., 2017). The increase in leak appears to occur despite no change or even a decrease in RyR expression (Yeh et al., 2008; Shanmugam et al., 2011; Brandenburg et al., 2016). Unlike in AF, the phosphorylation of RyRs does not appear to change in heart failure (Yeh et al., 2008; Brandenburg et al., 2016), and the increased leak is therefore likely to be caused by the increased atrial SR calcium content generally reported in large mammals with heart failure (Yeh et al., 2008; Clarke et al., 2015; Aistrup et al., 2017). As opposed to AF, atrial t-tubule loss in heart failure may facilitate calcium dependant arrhythmias by “orphaning” RyRs, promoting heterogenous calcium release and facilitating atrial alternans as has observed in the ventricle (Louch et al., 2006; Song et al., 2006; Heinzel et al., 2008; Li Q. et al., 2012; Nivala et al., 2015). It is noteworthy that atrial t-tubule loss in heart failure appears more severe than that observed in AF [at least in the sheep (Dibb et al., 2009; Lenaerts et al., 2009)] although whether the greater loss of t-tubules in heart failure increases the likelihood of AF remains to be determined.

In heart failure the amplitude of the calcium transient is important since impaired ventricular filling results in a greater reliance on atrial contraction (Kono et al., 1992). While it is clear that SR function is remodeled in heart failure, how decreased *I*_Ca(L)_ and increased SR calcium content interact to give rise to the systolic calcium transient is unclear, with both increased and decreased calcium transient amplitude reported (Table 1). One discrepancy may lie in experimental conditions. Under voltage clamp conditions decreased *I*_Ca(L)_ may decrease calcium transient amplitude (Clarke et al., 2015) whereas prolongation of the heart failure atrial action potential in current clamp recordings (Koumi et al., 1994) would be expected to promote *I*_Ca(L)_ or reverse mode *I*_NCX_ which together with increased SR calcium content could increase calcium transient amplitude (Yeh et al., 2008). This effect however does not explain all discrepancies.

Compounding the increased SR calcium content, atrial NCX activity generally increases in heart failure (Li et al., 2000; Cha et al., 2004a,b; Hohendanner et al., 2015, 2016) although some have found otherwise (Clarke et al., 2015). The combination of elevated SR calcium content and increased NCX favors the generation of delayed afterdepolarizations, and an increase in atrial afterdepolarizations in heart failure is indeed consistently reported (Yeh et al., 2008; Hohendanner et al., 2015, 2016; Aistrup et al., 2017), along with a higher propensity for these calcium waves to trigger action potentials (Hohendanner et al., 2016) potentially leading to re-initiation of AF.

#### Atrial stretch may mediate the changes in calcium cycling

One of the mechanisms by which heart failure induces changes in atrial calcium cycling is the mechanical stress placed on the atrial wall, caused by the increase in left ventricular end diastolic pressure elevating left atrial pressure. This mechanical stress takes two forms, and both have important effects on calcium cycling. Stretch is the result of tension placed on the atrial myocyte as the left atrium dilates, while shear stress results from elevated pressure in the atrium deforming the myocyte (Schonleitner et al., 2017).

Atrial stretch increases contractility acutely under physiological conditions and is commonly known as the Frank-Starling mechanism [as reviewed in Sequeira et al. (Sequeira and van der Velden, 2015)]. This rapid increase in contractile force is due to an increase in the myofilament calcium sensitivity and was originally described in the ventricle (Allen and Kurihara, 1982; Hibberd and Jewell, 1982) although the same mechanism has been shown to occur in the atria (Tavi et al., 1998). A second, slower increase in contractility also occurs in response to myocardial stretch and is known as the slow force response (Parmley and Chuck, 1973; von Lewinski et al., 2004). Much of the initial work characterizing this phenomenon focused on the ventricle and showed it was brought about by an increase in the calcium transient (Alvarez et al., 1999), whereby myocyte stretch activates the sodium-proton exchanger increasing intracellular sodium providing a gradient to increase calcium influx via reverse NCX (Alvarez et al., 1999; von Lewinski et al., 2003; Kockskamper et al., 2008). In ventricular myocytes, this is associated with an increase in the frequency of sparks (Iribe and Kohl, 2008; Iribe et al., 2009) with one potential mechanism being via enhanced ROS production leading to increased open probability of RyRs (Prosser et al., 2013). This NCX dependant mechanism however has not been shown to occur in the atria, with instead a dependence on angiotensin II and endothelin-1 signaling (Kockskamper et al., 2008).

Both acute (Bode et al., 2000; Eijsbouts et al., 2003; Kuijpers et al., 2011) and chronic (Solti et al., 1989) atrial stretch have been shown to promote AF and are comprehensively reviewed in Eckstein et al. (2008) and Nazir and Lab (1996). One of the better described mechanisms of AF vulnerability in chronic atrial stretch is dependent upon endothelin-1 signaling [as reviewed in Drawnel et al (Drawnel et al., 2013)]. Endothelin-1 concentrations in the left atrium increase in both AF and heart failure (Mayyas et al., 2010) and this may act locally to enhance diastolic leak and ectopy via inositol 1,4,5-triphosphate signaling (Proven et al., 2006; Tinker et al., 2016), as well as by upregulating profibrotic pathways implicated in structural remodeling (Ruwhof and van der Laarse, 2000; Burstein et al., 2008).

Atrial shear stress has also been shown to increase calcium sparks and waves (Woo et al., 2007; Kim and Woo, 2015; Son et al., 2016), but is less well studied than stretch. It is also unclear whether stretch and shear stress can produce additive effects on calcium cycling. Although the increase in calcium sparks is not believed to be pro-arrhythmic in the absence of structural heart disease (Schonleitner et al., 2017), the greater degree of stretch in heart failure compounded by the loss of atrial t-tubules (Dibb et al., 2009) may contribute to an increased frequency of afterdepolarizations. For comprehensive reviews of atrial stretch and shear stress see Thanigaimani et al. (2017) or Ravelli (2003).

#### Impaired energy supply may also contribute to the changes in calcium cycling and ion channel homeostasis

Heart failure may also cause changes in atrial calcium cycling through mitochondrial dysfunction. Mitochondrial dysfunction, increased ROS production and increased oxidative stress occur in heart failure and have been extensively reviewed elsewhere (Dorn and Maack, 2013; Bertero and Maack, 2018). The mitochondrial dysfunction that occurs in failing ventricular myocytes also occurs in the atria of those with heart failure (Cha et al., 2003; Marin-Garcia et al., 2009). This results in a mismatch of energy demand and supply within the myocyte and necessitates a change in cellular metabolism to try to correct this (Fukushima et al., 2015). There is evidence in both heart failure and AF that myocytes undergo a change in transcription of key metabolic and cell signaling pathways (Barth et al., 2011, 2016). The downregulation of the metabolic pathways correlates with a reduction in transcription of calcium cycling proteins such as the LTCC, RyR, SERCA, and PLB, which suggests the metabolic shift in response to an energy deficit is associated with changes in calcium cycling although a direct cause has not been established (Barth et al., 2016). The upregulation of calcium-dependent signaling pathways such as the Wnt pathway may encourage further structural remodeling to occur to support AF (Barth et al., 2011).

#### Pulmonary vein firing accelerates in heart failure

In addition to its effects on the atrial myocardium, heart failure also influences the pulmonary veins, potentially increasing their spontaneous firing rate. Increasing intra-atrial pressure, as found in heart failure, accelerates pulmonary vein firing in intact sheep hearts (Kalifa et al., 2003). While this increase appears to be mediated by changes in calcium cycling leading to more delayed afterdepolarizations (Chang et al., 2008; Loh et al., 2009; Lin et al., 2016), the cellular changes responsible for this are unclear. While increased levels of circulating B-type natriuretic peptide have been suggested to increase the rate of spontaneous firing due to increased *I*_Ca(L)_ and *I*_NCX_ but decreased late *I*_Na_ (Lin et al., 2016), others report an increase in DADs in the setting of heart failure with decreased *I*_Ca(L)_ and increased late *I*_Na_ (Chang et al., 2008). Overall, heart failure appears to increase the rate of pulmonary vein firing, but the discordant results and lack of studies in large mammals make the precise mechanism underlying this unclear.

#### The effects of heart failure on the atrial action potential are unclear

Heart failure has been shown to affect atrial electrophysiology although the current evidence is inconsistent. Some describe a prolongation of the atrial action potential in heart failure (Koumi et al., 1994; Li et al., 2000; Yeh et al., 2008) associated with prolongation of the refractory period, which has been suggested to promote AF by facilitating early afterdepolarizations. However, others have described shorter action potentials and refractory periods in association with heart failure (Koumi et al., 1997; Schreieck et al., 2000; Sridhar et al., 2009; Workman et al., 2009; Clarke et al., 2015) and argued that this might facilitate AF by decreasing the wavelength of re-entrant circuits allowing a greater number to co-exist. While experimental differences between studies such as species, rate of stimulation, stage of heart failure and level of t-tubule loss likely contribute to the disparate literature this suggests that the influence of heart failure on action potential duration and refractory period may be of secondary importance in the initiation of AF. This is supported by evidence from models of heart failure that have not found any difference in refractory period but still shown markedly increased vulnerability to AF (Li et al., 1999), and from tachy-paced models of heart failure that have been allowed to recover demonstrating that increased vulnerability to AF remains despite the refractory period returning to baseline (Cha et al., 2004b).

#### Heart failure promotes structural remodeling of the atria

A major determinant of the susceptibility to AF in those with heart failure is remodeling of the atrial structure in the form of atrial dilatation (Sridhar et al., 2009; Melenovsky et al., 2015) and fibrosis (Li et al., 1999; Shinagawa et al., 2002; Cha et al., 2004a,b; Todd et al., 2004). While similar structural changes can occur in AF, the structural remodeling associated with heart failure is more extensive and progresses more rapidly than is seen as a response to AF (Li et al., 1999). Macroscopically, this remodeling takes the form of left atrial dilatation, which regardless of its underlying cause is associated with an increased risk of developing AF (Solti et al., 1989; Huang et al., 2003) and can be used clinically to predict the success of treatments aimed at restoring sinus rhythm (Brodsky et al., 1989; Helms et al., 2009; Montefusco et al., 2010). In heart failure, this dilatation occurs due to a rise in left ventricular end diastolic pressure (Melenovsky et al., 2015). At a microscopic scale, the dilatation is accompanied by atrial fibrosis. The atrial fibrosis induced by heart failure leads to slower, heterogeneous conduction promoting wavebreak and re-entry (Li et al., 1999; Sanders et al., 2003; Akkaya et al., 2013). In an effort to prevent the deleterious effects of structural remodeling, anti-fibrotic agents have been explored and shown to reduce atrial fibrosis and vulnerability to AF in models of heart failure (Lee et al., 2006; Le Grand et al., 2014) and appear to reduce the incidence of AF in clinical trials (Chaugai et al., 2016).

### How does atrial fibrillation exacerbate heart failure?

When AF develops in those with heart failure it is associated with worsening symptoms and prognosis, in part due to a loss of atrial contraction further impairing ventricular filling. However, other factors influencing the worsening of heart failure include rapid heart rates and an irregular ventricular response compromising ventricular performance, atrial stunning, and valvular dysfunction, mediated in part by alterations in both atrial and ventricular calcium handling.

#### Cardiac output is impaired during atrial fibrillation

In sinus rhythm, atrial contraction contributes to ventricular filling. Loss of meaningful atrial contraction during AF can reduce cardiac output by up to 25% (Hecht and Lange, 1956; Naito et al., 1983). Many of those with heart failure are less able to increase their cardiac output in response to exercise (Mc et al., 1939), or may even have a lower cardiac output at rest (Stead et al., 1948), and the additional loss from AF exacerbates symptoms such as breathlessness and exercise tolerance (Ponikowski et al., 2016).

In addition to the loss of atrial contractility, the rapid ventricular rates which frequently occur in untreated AF reduce ventricular diastolic filling compared to a physiological heart rate (Raymond et al., 1998), further decreasing cardiac output. If these episodes of rapid ventricular rates are prolonged, they can impair ventricular function, although this can be reversed by restoring sinus rhythm or by slowing the ventricular rate in AF (Khan et al., 2008). The impaired ventricular function may be a primary cause for heart failure (a tachycardia-induced cardiomyopathy), or may be appreciated as a worsening of left ventricular function that is already impaired for another reason (Grogan et al., 1992; Fujino et al., 2007; Simantirakis et al., 2012). Rapid cardiac stimulation reliably induces dilated cardiomyopathy and end stage heart failure and is therefore commonly used as an experimental model of heart failure (Li et al., 2000; He et al., 2001; Cha et al., 2004a; Clarke et al., 2015). In this context, impaired ventricular systolic function is associated with remodeling of calcium handling—the high ventricular rates lead to t-tubule loss and a decreased ventricular calcium transient amplitude which can arise by a decrease in either SR calcium content or peak *I*_Ca(L)_ (He et al., 2001; Hobai and O'Rourke, 2001; Briston et al., 2011). It seems likely that the effects of tachycardia (as a result of AF) on ventricular function seen clinically may be mediated by a similar mechanism although these animal models do not take into account any change in rhythm which can occur in AF.

AF also results in an irregular ventricular response which has compounding effects on cardiac output. Short-term irregularity in ventricular contraction is associated with lower cardiac output independently of heart rate in dogs (Naito et al., 1983), and impaired systolic function in man (Sramko et al., 2016). The effects of an irregular ventricular rate may develop further over time driven by changes in calcium handling, as ventricular myocytes paced in an irregular rhythm for 24 h demonstrate lower calcium transient amplitude, reduced expression of SERCA, and reduced phosphorylation of phospholamban compared to control (Ling et al., 2012).

AF is also associated with the development of valve dysfunction in the form of mitral regurgitation (Skinner et al., 1964). While mitral regurgitation can affect atrial structure in ways which promote AF, AF also appears to promote mitral regurgitation (Liang and Silvestry, 2016). The irregular ventricular response acutely influences mitral regurgitation (Naito et al., 1983), while in the longer term, persistent AF causes dilatation of the mitral valve annulus leading to functional mitral regurgitation (Liang and Silvestry, 2016). Mitral regurgitation contributes to the increase in pulmonary capillary wedge pressure associated with AF (Clark et al., 1997) which leads to fluid leaking from the pulmonary capillaries to cause pulmonary oedema giving rise to breathlessness.

Overall, the decrease in cardiac output due to a combination of: loss of atrial function, loss of regular ventricular contraction and increased mitral regurgitation can exacerbate pre-existing heart failure.

#### Atrial function remains depressed after atrial fibrillation has terminated

Atrial function remains temporarily depressed following the restoration of sinus rhythm in patients who have periods of AF, known as atrial stunning (Schotten et al., 2003a). This effect is unsurprising, as the changes in calcium handling that take place during AF which lead to decreased calcium transient amplitude and hence decreased contractility require time to resolve (Schotten et al., 2002; Lenaerts et al., 2009; Voigt et al., 2012). The effects of stunning can last for up to a month, and the time to recovery is proportional to the duration of AF prior to cardioversion (Harjai et al., 1997; Sparks et al., 1999). It appears that a major determinant of the residual decreased contractility seen in stunned atrial myocytes is decreased *I*_Ca(L)_, although pathological remodeling of contractile proteins contributes to this process (Schotten et al., 2001). While electrical reverse remodeling can occur between short bouts of AF (Todd et al., 2004), it is possible that the structural remodeling that occurs in persistent AF may have an irreversible effect on atrial contraction despite restoration of sinus rhythm, but evidence for this is lacking.

## Is the pathophysiology of atrial fibrillation in heart failure different to atrial fibrillation in the absence of structural heart disease?

AF can also develop in the absence of heart failure, and one subtype of AF which has historically been described in the literature is that of lone AF. “Lone AF,” defined by the absence of overt heart disease or precipitating illness is relatively unusual, accounting for only 10% of new diagnoses of AF (Kim et al., 2016). Some argue that even this figure may be an overestimate as more thorough investigation might reveal underlying disease in many of these patients (Wyse et al., 2014). Even in the absence of overt heart disease, many young patients who develop AF may still have an underlying cause in the form of a genetic predisposition caused by variants in ion channels (Weng et al., 2017). We recognize the term “lone AF” is controversial based on inconsistencies in the definition and a lack of a distinct pathophysiological mechanism (Potpara and Lip, 2014; Wyse et al., 2014). For that reason, we have compared AF that occurs on the background of heart failure vs. AF in the absence of structural heart disease or a major risk factor.

The abnormalities in atrial calcium handling that occur in response to heart failure have some similarities with those that occur in AF (Table 1). The decrease in atrial *I*_Ca(L)_ in heart failure mirrors that seen in both paroxysmal and persistent AF. SR calcium content increases in heart failure as some studies report in early or paroxysmal AF although this is not found in persistent AF. There is limited data on the effects of heart failure on atrial RyRs but, unlike in AF, heart failure does do not appear to lead to RyR phosphorylation. However, the net effect of these modifications leads to a similar endpoint of increased RyR leak and afterdepolarizations in heart failure and AF. Although the pulmonary veins are the source of triggers for AF in the majority of patients with lone AF (Haissaguerre et al., 1998), the remodeling of atrial calcium cycling that promotes afterdepolarizations throughout the atria theoretically increases the chance of triggers occurring from non-pulmonary vein sources, potentially diminishing the relative importance of the pulmonary veins in patients with heart failure. This may be of significant practical importance as it may influence the ablation strategies used in clinical practice which typically focus on the pulmonary veins, and may contribute to the lower success rates of ablation described in those with heart failure (Anselmino et al., 2014).

A major difference between the AF that occurs alongside heart failure and the early stages of AF that occurs without evidence of overt heart disease is the atrial structure. As we have seen, heart failure leads to atrial dilatation and fibrosis, promoting heterogeneous conduction, wavebreak, and a substrate that supports multiple simultaneous re-entrant circuits. The atrial structural remodeling found in heart failure occurs more rapidly and to a greater extent than in AF without heart failure (Li et al., 1999). This appears to be an important determinant of atrial vulnerability in canine models of heart failure, in which far longer durations of AF were observed in dogs with heart failure compared to control animals (Li et al., 1999). This experimental work has parallels with epidemiological studies in man. Patients with heart failure are less likely to have a paroxysmal form of the disease and more likely to have persistent or permanent AF than those without heart failure (Silva-Cardoso et al., 2013). Although this data does not describe the initial presentation of patients with AF, it is possible that while the natural history of lone AF typically begins with paroxysmal AF in structurally normal atria, in those with heart failure AF may be more likely to present with persistent AF. Structural remodeling of the atria is associated with a lower chance of maintaining sinus rhythm in response to medication, cardioversion, or ablation when assessed using left atrial diameter e.g., (Nedios et al., 2015) or fibrosis assessed by MRI scanning (Marrouche et al., 2014). Maintaining sinus rhythm is accordingly less likely in those with heart failure than those without (Anselmino et al., 2014; Fredersdorf et al., 2014). Even though these structural changes occur more rapidly in heart failure, atrial dilatation and fibrosis also occur in advanced forms of AF in those without heart failure (Schotten et al., 2003a).

Overall, the pathophysiology of AF in those with heart failure shares many similarities to the advanced AF seen in those without heart failure. Heart failure patients often present with AF some distance along their journey of atrial remodeling.

## What are the limitations of research in understanding the pathophysiology of atrial fibrillation?

Although there is strong evidence to support the role of calcium remodeling as a whole in AF and heart failure, some aspects of this process show wide variation in the experimental data which often produces conflicting results. There are a number of potential reasons for the differences in findings, and herein we provide a critical review of these.

### Studies of human myocytes are hampered by comorbid conditions and medications

Obtaining human atrial myocytes to study calcium handling can be performed by using samples of atrial appendage that are routinely discarded following cardiac surgery (Voigt et al., 2015). While patient samples offer a unique opportunity to study the disease in which we are interested this approach suffers from several disadvantages. Research participants need to have an indication for cardiac surgery and therefore have other cardiovascular conditions that confound interpretation of results. Common indications for surgery include coronary artery bypass grafting for ischaemic heart disease and valve replacement for mitral valve disease, both of which are independently associated with structural remodeling of the atria and an increased vulnerability to AF. Furthermore, these patients are often taking medications that influence cellular electrophysiology and calcium handling such as beta-adrenoceptor blockers. While atrial tissue would ideally be obtained from humans without pre-existing cardiac disease, access to this tissue is extremely limited as the risks associated with an atrial biopsy far outweigh any potential benefit (From et al., 2011).

### Some animal models may not be fully representative of human atrial fibrillation

To avoid the limitations of using human tissue, animal models are frequently used which include rodents and large mammals such as dogs, sheep, or goats. However, spontaneously occurring AF in these animal models is rare (Heijman et al., 2016) and an artificial trigger is required. The artificial trigger is often in the form of rapid atrial pacing which mimics pulmonary vein triggers (Morillo et al., 1995), although other models such as vagal stimulation are also used and are reviewed further by Nishida et al. (2010). These models have been helpful in characterizing the early steps in the development of AF, as episodes of AF early on in the disease course in humans may occur infrequently and take months or years to diagnose (Kirchhof et al., 2016).

Animal models of AF benefit from the fact that the frequency and duration of arrhythmia can be quantified allowing a time course of remodeling to be determined. However, it is uncertain whether the atrial changes induced artificially by rapid atrial pacing which produce persistent AF within days to weeks are truly comparable to the human condition in which persistent AF typically develops over months to years. Furthermore, the atrial arrhythmias seen in rodents may be a poor comparator for human AF due to the differences in ion channel expression between rodents and man (Gussak et al., 2000), the differences in the relative importance of SERCA and NCX in clearing cytosolic calcium (Bassani et al., 1994), and the lack of an extensive t-tubule network in rodents that is found in larger mammals (Richards et al., 2011). On the other hand genetic manipulation of rodents can result in powerful models for understanding arrhythmic mechanisms.

The results from animal models of atrial electrophysiology in heart failure may also be confounded by the technique used to induce ventricular dysfunction. Many large animal models use ventricular tachypacing to produce a dilated cardiomyopathy. While this technique is likely to be representative of end stage heart failure, it must be borne in mind that tachycardiompathy accounts for only a minority of the causes of heart failure seen in humans (Mosterd and Hoes, 2007) and may therefore differ in important regards from more representative aetiologies of heart failure such as ischaemic heart disease. For example, ventricular tachypacing may inadvertently cause rapid atrial pacing via retrograde conduction through the atrioventricular node, mimicking AF. It is therefore important that this potential confounder is pre-empted, measured, and if necessary addressed by blocking the AV node (Li et al., 1999).

Overall, interpreting the results of studies of calcium handling in AF requires careful attention to the methods used including species, duration of AF, and confounding comorbidities and medications.

## Conclusions

Calcium plays a fundamental role in the pathophysiology of AF, as well as in the bidirectional relationship between AF and heart failure. Remodeling of calcium cycling in response to rapid atrial stimulation can be seen within days in the form of calcium signaling silencing. However, these initially protective changes eventually become pathological as paroxysmal AF develops and can facilitate the progression to persistent AF. A recurring theme throughout this progression is increased diastolic leak of calcium from RyRs which promotes afterdepolarizations. The rise in intracellular calcium concentrations also activates pro-fibrotic pathways leading to structural remodeling in the atria that produces a substrate that can support the complex re-entry seen in AF. This review has provided a comprehensive stepwise description of the fundamental aspects of calcium cycling remodeling.

Heart failure leads to remodeling of calcium cycling and structural changes within the atria which support persistent AF. In return, AF exacerbates heart failure by reducing cardiac output, due in part to a loss of atrial contraction but also by affecting ventricular calcium cycling. A vicious circle ensues whereby one condition begets the other. Questions remain surrounding the complex interaction between AF and heart failure, but the undeniable importance of calcium cycling remodeling in both conditions should prompt the exploration of these mechanisms as potential novel therapeutic targets.

## Author contributions

ND prepared the first draft of the manuscript, which was critically revised by CP, JC, GM, DE, AT, and KD. JC produced the figures. All authors agree with its final content.

### Conflict of interest statement

The authors declare that the research was conducted in the absence of any commercial or financial relationships that could be construed as a potential conflict of interest.
